# Routine early post-operative X-ray following internal fixation of intertrochanteric femoral fractures is unjustified: a quality improvement study

**DOI:** 10.1186/s13018-018-0896-9

**Published:** 2018-07-31

**Authors:** David Segal, Ezequiel Palmanovich, Ali Faour, Elad Marom, Viktor Feldman, Eyal Yaacobi, Omer Slevin, Benjamin Kish, Yaron S. Brin

**Affiliations:** Orthopedic Department, Meir Medical Center, 59 Tschernihovsky St., 44281 Kfar Saba, Israel

**Keywords:** Post-operative radiography, Intertrochanteric, Check X-ray, Follow-up

## Abstract

**Background:**

There is no consensus regarding the proper radiographic protocol following closed or open reduction and internal fixation for intertrochanteric femoral fractures. The objective of this study was to assess the role of early postoperative imaging studies when deciding about weight bear limitations and reoperations.

**Methods:**

A prospective cohort study of 100 patients (26 men and 74 women, at a mean age of 79.8 years) treated by closed or open reduction and internal fixation for AO31A fractures was conducted. According to the AO classification, there were 25 cases of 31A1, 54 cases of 31A2, and 21 cases of 31A3. For every patient, the intraoperative fluoroscopy studies were recorded and post-operative radiograms were taken during the first week. Excluded were patients for whom the early X-rays were clinically indicated. The intraoperative AP and axial fluoroscopy studies were compared with the radiograms taken during the first post-operative week. The investigators compared the decisions regarding weight-bearing limitations and the need for re-operation before and after conducting the radiograms.

**Results:**

The early post-operative imaging studies did not change weight-bearing limitations nor did they lead to consecutive surgical treatments.

**Conclusions:**

Unless indicated by physical examination, there is no value to routine post-operative radiograms within the first few days after closed reduction and internal fixation of intertrochanteric femoral fractures with regard to weight-bearing limitations and re-operation decisions.

**Trial registration:**

Identifier: NCT02868125.

## Background

The incidence of intertrochanteric femoral fractures (IFF) is very high [1, 2]. The primary treatment modality for these fractures is closed reduction and internal fixation (CRIF) with an intramedullary nail (IMN) or a dynamic hip screw (DHS) [1, 2]. These operations are conducted with the aid of intra-operative fluoroscopy in order to confirm that the bone structure is restored and the fixating device is properly positioned. Recording the anterior-posterior (AP) and axial fluoroscopic images that show the reduced fixated femur is a common practice.

Early postoperative imaging (EPI) studies are used to better verify that the implant was well placed, that the fracture was reduced appropriately, and that proper imaging studies are recorded electronically [3, 4]. There is no consensus regarding the proper timing for the first post-operative radiography studies for patients that undergone CRIF of IFF. A survey we conducted among orthopedic surgery specialists in nine university hospitals in Israel revealed that three perform the first X-ray imaging study on the first post-operative day, one each on the first and second post-operative week, and the remaining four at the sixth week. This lacks consensus. While the value of EPI for other fracture fixations was previously questioned by Canadian researchers [3], we found lack of prospectively investigated data regarding EPI of patients with IFF that were operated by IMN. A few studies from the USA and Europe that dealt with the value of EPI in IFF [5–9] did reflect an international eager to limit the execution of these radiographs but have included mainly patients that were operated with DHS and hemiarthroplasties and only few IMN patients, the current work-horse in this field. Evidence-based data is therefore needed in order to allow surgeons worldwide to plan the proper imaging protocol for IMN patients.

The objective of this study was to assess the effect of routine first week postoperative imaging studies for patients operated by IMN or DHS due to an IFF on the weight-bearing restrictions and decisions for re-operations. The investigators’ hypothesis was that routine EPI will have no effect on the weight-bearing restrictions and reoperation decisions.

## Methods

This prospective study was approved by the local institutional review board. Since the patients’ treatment and follow-up plans were not modulated by the research, and their inclusion did not add any risks, a waiver of consent was approved. Patients of all ages who were treated in our institution between May and September 2016 at a level 1 trauma center in Israel by a closed or open reduction and internal fixation due to IFF (AO-31A) were enrolled. The study was planned for 12 months but was stopped after the inclusion of 100 patients due to conclusive results. Excluded were patients with pathological post-operative clinical findings, such as mal-rotation on post-operative physical examination or a significant hip pain that led to a radiographic work-up that was not part of routine follow-up. These patients were not part of the study population and were not followed. Patients for whom proper fluoroscopy imaging studies were not recorded were also excluded. We found it essential to define weight-bearing allowance, which was the main outcome measure in this study even for the few preoperatively non-ambulatory patients in order to assess the need for EPI in this group as well, and to make a decision that was focused on the fracture-implant radiographic appearance. Eventually, this small group had a minimal impact on the study outcomes. For each patient, three sets of imaging studies were taken in accordance with the department’s protocol: (1) Upon presentation, a pelvis AP and hip AP and orthogonal X-ray studies were taken as part of the emergency department work-up. (2) Intra-operative AP and orthogonal fluoroscopy images were recorded after the fracture was reduced and fixated. (3) On the third to sixth postoperative days, every patient underwent an AP and an orthogonal X-ray imaging study of their operated hip. This study was taken in the radiology department, and its execution on the third, fourth, fifth, or sixth day relied on the availability of the radiography center. All studies were assessed for both their quality evaluation and for radiographic measurements. The fracture types were determined by three authors (DS, BK, YB) 1 day following the operation based on the admission X-ray radiographs, according to the Arbeitsgemeinschaft fur Osteosynthesefragen/Orthopedic Trauma Association (AO/OTA) classification system [10].

Post-operatively, weight-bearing restrictions and the need for reoperation were determined based on the patient’s primary medical and physical status, fracture type, possible surgical complications, and reduction and the fixation quality (as will be further elaborated) at three stages: (1) immediately after the operation, by the surgeon, based on the fluoroscopy studies; (2) 1 day following the operation, by the departmental review board, based on fluoroscopy studies; and (3) 1 week following the operation, by the departmental review board, based on all the mentioned information plus the EPI. On the second and third stages, the same group of surgeons made the decisions according to the same criteria that were noted on stage 1 and the only added information at the third stage was the EPI. Therefore, by comparing these two consecutive stages, the investigators aimed to reveal the additive value of the last study. Possible instructions were full, partial, touch-down, or non-weight bearing. Reoperation was considered when significant implant or bone movement, malalignment, or medially protruding (intra-articular) lag screw were identified, and relatively discouraged when significant comorbidities existed. Weight-bearing restrictions were considered (although not categorically applied) when the fracture’s structure was unstable or when the lag screw’s tip was too superior or close to the subchondral bone (as might be implied by a low tip-apex distance (TAD) value or a superior screw location).

While reviewing the radiographs, the neck-shaft angle, the tip apex distance, and the fixator location (with the femoral neck divided to nine zones on the coronal plane [11]) were measured by one of two investigators (DS or AP) both on the intraoperative fluoroscopy images and on the EPI studies using the PACS software )Carestream Health, Inc.) in order to more accurately assess changes in the bone-device construct. Proper fixation was defined when neck-shaft angle was ≥ 130° [4, 12–14], when tip apex distance was in the range of 20–25 mm [14–19], and when the lag screw was located center-center or center-inferior [4, 11, 15, 17, 20].

### Statistical analysis

The correlation between fluoroscopy and EPI was evaluated with a kappa test, with a value ≥ 0.75 considered excellent. The differences between the groups in which EPIs were of value to the unaffected group were conducted using a uni-variant analysis according to the variants nature: chi-square test analysis and its derivatives for nominal variants and Fisher exact *t* test for consecutive variables. A power analysis found that a sample size of seven patients in each group (14 altogether) was needed in order to reveal a difference of 5° of the mean NSA (the mean change in operated unstable IFF [23]) between the two groups with a power of 0.8 and an alpha of 0.05. A multi-variate analysis was conducted for statistically significant differences. The number needed to treat was calculated in order to understand how many patients need to undergo an EPI in order to influence the decision on one patient.

## Results

Of 117 patients enrolled in the study, 17 were excluded, 10 due to lack of recorded intraoperative fluoroscopy studies; 3 were discharged without post-operative radiographic studies; 3 were radiographed not as part of a routine follow-up (2 due to post-operative pathological findings on physical examination (external rotation of the operated hip) and 1 due to radiographic signs of significant mal-reduction on fluoroscopy studies that were suspected cause instability); and 1 patient died 2 days after surgery. Ultimately, 100 patients with proper routine postoperative X-ray studies were included (Table 1).

**Table 1:** Cohort characteristics

Characteristic	Cohort (*N* = 100)
Age	Mean = 79.8 years
Sex	
Men	26
Women	74
Pre-operative ambulation	
Independent	49
Cane-assisted	13
Walker-assisted	29
Wheelchair	1
Bed ridden	5
Fracture type	
AO31A1	25
AO31A2	54
AO31A3	21
Fixation type^c^	
Dynamic hip screw	16
Intramedullary nail	84
Gamma3	62
PFT	4
Zimmer	9
PFNA	9
TAD^a^	21.63 mm (SD = 3.53)
NSA^b^	129.29° (SD = 5.89)
Fixator location	
Center-center	81
Center-inferior	16
Posterior-inferior	2
Posterior-center	1

^a^*TAD* tip apex distance

^b^*NSA* neck shaft angle

^c^Fixation devices: Gamma3® (Stryker, MI); PFNA® (DePuy Synthes, PA); Targon® PFT/PF, (Aesculap, PA); Zimmer’s nail®, (Zimmer, IN); Dynamic hip screw (Smith & Nephew, Memphis, TN)

Following imaging study evaluation, all patients (except for 1 who was restricted to partial weight-bearing) were allowed full-weight bearing and 92 performed ambulation with a stance on the operated leg before EPI. The seven patients who did not bear weight were preoperatively bedridden patients and wheelchair-dependent (who were obviously not expected to walk) and one walker-dependent patient who did not cooperate with physical therapy due to a general weakness that has resolved only after the EPI was taken. Information gathered from the EPI studies did not influence weight-bearing restrictions or lead to reoperation in any of the cases. During the study, the surgeons admitted that the images did not offer any new perception, and since the results were clear by the 100 patients, the investigators decided to end the study.

Based on the intraoperative fluoroscopy images, the mean neck-shaft angle (NSA) was 129.29° (SD = 5.89) and the mean tip apex distance (TAD) was measured as 21.63 mm (SD = 3.53). Eighty-one fixators were placed in a center-center position, 16 were center-inferior, 2 were posterior-inferior, and 1 was posterior-center. The department review board that included most authors (DS, AF, OS, EP, BK, and YB) found that only eight EPI studies were both true AP and proper orthogonal. Accordingly, it was not possible to measure NSA and TAD in the vast majority of the EPIs and to objectively compare them to the fluoroscopy findings.

As there were no patients whose treatment was influenced by EPI findings, the statistical analysis was abandoned.

## Discussion

Determining the correct follow-up radiography protocol is important and should be directed by clear objectives. When imaging fixated fractures, the surgeon initially aims to assess reduction maintenance and implant position. Later imaging studies aim to help in assessing fracture union or recognizing pathological ossified tissue formation. Understanding the timeline and risk factors of structural postoperative complications is key factors to properly plan the follow-up radiography protocol for each patient.

While repaired fracture fragments move slightly during the first few postoperative months, partially due to the desired compression mechanism, only few patients actually suffer from symptoms related to fixation failures that lead to revision surgery [12, 21]. A few researchers studied structural changes in the bone-device construct and found expected movement during the first 6 weeks following surgery. Gardner et al. studied the radiographic outcomes of 97 intertrochanteric hip fractures treated with trochanteric helical blade fixation nail [22]. The mean telescoping was 4.3 mm in the unstable fracture group compared with 2.6 mm in the stable group. Blade migration within the femoral head averaged 2.2 mm overall. The important changes related to telescoping and blade migration occurred during the first 6 weeks. Parajian et al. found a mean decrease of 4.6° in NSA for unstable IFF treated with either dynamic hip screws or proximal femoral nail [23]. Two studies showed that fixation failures were diagnosed at a mean of 5.2–12 weeks [14, 15]. As these findings amplify the need for postoperative imaging studies in order to make sure that the reduction was maintained, the fact that changes occur only weeks to months after surgery put the need for first week follow-up radiographies in question.

Studies regarding the utility of EPI as part of the patient follow-up after internal fixation of a wide range of fractures, including proximal femur fractures, are scarce [5–9, 24]. Although the use of IMN for IFF has become the work-horse in this field, the main modality of treatment addressed in the previous literature was DHS, with no sufficient data on IMNs. All authors have conducted retrospective studies and have suggested that routine “check X-rays” during the first post-operative days should be avoided. It was also shown that orthopedic surgeons were generally amenable to more liberal post-operative imaging protocols [3].

Routine EPIs for patients with IFF are disadvantageous from both economical and medical perspectives. The estimated accumulated cost of imaging every patient following IFF CRIF during their hospitalization includes radiography facility time, hospital porters’ time, orthopedic ward coordination which translates to nurses’ and surgeons’ working time, and in some cases, prolonged hospitalization in order to complete the required images. In the investigators’ hospital, the accumulative costs per year were equivalent to at least 10 radiography facility work-days and 350 porter hours. The radiation burden is composed of AP and lateral hip X-rays that expose the patient to 0.7–0.8 mSv, equivalent to about 40 chest X-rays [5, 25]. Patients pain and discomfort due to transfer to the radiography center and back is immeasurable, but obvious.

Both current and prior investigators did not find value in conducting EPI studies for patients who underwent IFF CRIF, even when the fractures were defined pre-operatively as unstable or the fixations were sub-optimal [15, 22, 26]. Furthermore, the current study found that too early radiographs did not provide new information and were of low quality because it was difficult for patients to cooperate with positioning on the radiography table due to post-operative pain and discomfort.

Relying on previous literature, it is suggested that the proper timing for the first routine hip joint radiograph is 6 weeks after IFF CRIF [5–9, 11, 12, 14, 15, 17, 21, 22, 27]. At this point, most compression and telescoping dynamics have occurred and the final bone-implant length and structure, along with radiographic signs of fracture union or possible failure, can be evaluated and documented.

**Fig. 1:**
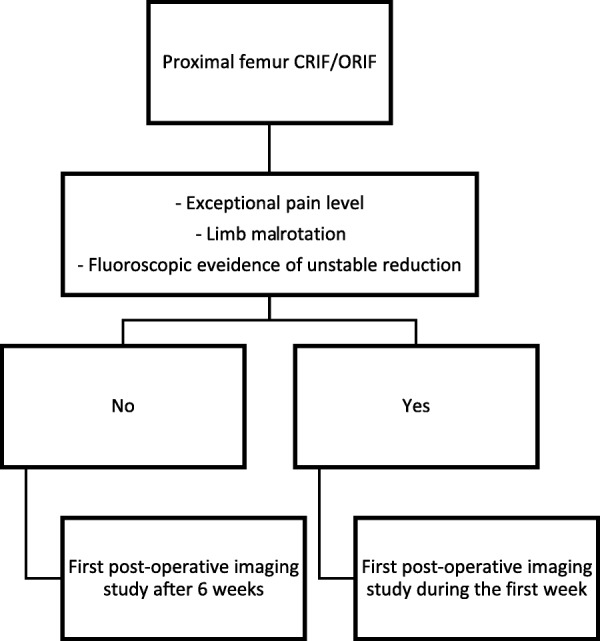
Post-operative imaging protocol following CRIF/ORIF of IFF

The focus of this study was routine EPI studies. Accordingly, patients who had encountered extreme hip pain during the first post-operative days or exhibited lower limb mal-rotation were imaged as part of diagnostic work-up and were excluded from the study. In these cases, the surgeon had to rule out joint penetrating implant or fixation failure and collapse and to reassess the bone-implant construct. While these imaging studies were not part of the routine protocol, it is essential to note that these clinical situations indicate the need for an EPI, even though they were not studied in the current report. In order to determine the yield of EPI in this group of patients, a large series of symptomatic patients should be analyzed. A graphic illustration of our suggested protocol is presented in Fig. 1.

This study has a few weaknesses. As the investigators focused solely on the first post-operative week, a long-term follow-up was not presented. Patients were excluded from the study due to clinical findings. While physical examination findings were quantifiable, pain and discomfort evaluation was more subjective, even though not biased as the decision for exclusion was always done by the senior author (YB). Osteoporosis, which can influence the bone-implant stability, was not evaluated as part of the study. Since the exclusion criteria were liberal, we assumed that our cohort had represented the local age-specific population in that regard and was not evaluated. The measurements of the TAD, NSA, and fixator location were conducted without an inter- and intra-observer reliability assessment. As these measurements were eventually not helpful in the comparison of the intraoperative fluoroscopy studies and the EPI studies, we did not find in essential to insist on a few measurements for each image.

## Conclusion

Unless indicated by physical examination, there is no value to routine post-operative radiograms within the first few days after CRIF/ORIF of intertrochanteric femoral fractures with regard to weight-bearing limitations and re-operation decisions [6, 8, 9].

## Abbreviations

AP: Anterior-posterior
CRIF: Closed reduction and internal fixation
DHS: Dynamic hip screw
EPI: Early postoperative imaging
IFF: Intertrochanteric femoral fracture
IMN: Intramedullary nail
NSA: Neck-shaft angle
TAD: Tip apex distance
